# Evidence of supratentorial white matter injury prior to treatment in children with posterior fossa tumors using diffusion MRI

**DOI:** 10.1093/noajnl/vdaf053

**Published:** 2025-03-05

**Authors:** Emily R Drabek-Maunder, Jenny Gains, Darren R Hargrave, Kshitij Mankad, Kristian Aquilina, Jamie A Dean, Andrew Nisbet, Chris A Clark

**Affiliations:** Great Ormond Street Institute of Child Health, University College London, London, UK; Department of Medical Physics and Biomedical Engineering, University College London, London, UK; Great Ormond Street Hospital for Children, London, UK; University College London Hospitals NHS Foundation Trust, London, UK; Great Ormond Street Institute of Child Health, University College London, London, UK; Great Ormond Street Hospital for Children, London, UK; Great Ormond Street Institute of Child Health, University College London, London, UK; Great Ormond Street Hospital for Children, London, UK; Great Ormond Street Institute of Child Health, University College London, London, UK; Great Ormond Street Hospital for Children, London, UK; Institute for the Physics of Living Systems, University College London, London, UK; Department of Medical Physics and Biomedical Engineering, University College London, London, UK; Great Ormond Street Institute of Child Health, University College London, London, UK

**Keywords:** diffusion MRI, diffusion tensor imaging (DTI), neurite orientation dispersion diffusion imaging (NODDI), posterior fossa tumor, tract-based spatial statistics (TBSS)

## Abstract

**Background:**

Pediatric brain tumor survivors can have neurocognitive deficits that negatively impact their quality of life, but it is unclear if deficits are primarily caused by treatments, such as radiotherapy, or manifest earlier due to the tumor and related complications. The aim of this work is to characterize white matter injury caused by brain tumors, unrelated to treatment effects, and explore heterogeneity in these white matter abnormalities between individual patients.

**Methods:**

We used diffusion tensor imaging and neurite orientation dispersion diffusion imaging to assess white matter injury in 8 posterior fossa tumor patients. A novel one-against-many approach was used by comparing an individual patient to 20 age- and sex-matched healthy controls to assess variability in white matter abnormalities between the posterior fossa tumor patients. White matter was analyzed at presentation (prior to treatment), postsurgery (24–72 hours after surgery), and at follow-up (3–18 months after surgery).

**Results:**

We demonstrate white matter abnormalities in 5 posterior fossa tumor patients before treatment, likely related to tumor-induced hydrocephalus, which persisted after treatment. White matter changes were complex and patient-specific, and group-based comparisons with control subjects may fail to detect these individual abnormalities.

**Conclusions:**

Identifying pretreatment white matter injury in posterior fossa tumor patients highlights the importance of personalized assessment of brain microstructure, which should be considered in minimizing neurocognitive deficits to improve patient quality of life.

**Key Points**

- Supratentorial white matter injury found before treatment in posterior fossa tumor patients.
- White matter abnormalities are patient-specific and persist after treatment.
- Personalized brain assessment may help reduce neurocognitive deficits seen after treatment.

**Importance of the Study**

Neurocognitive deficits in pediatric brain tumor survivors may originate from the tumor itself rather than solely from treatment. Identifying pretreatment white matter abnormalities can help clarify the origins of these deficits. This is the first study to use diffusion MRI and a novel one-against-many approach to assess the white matter in 8 individual posterior fossa tumor patients prior to treatment. Pretreatment white matter abnormalities, likely related to tumor-induced hydrocephalus, were found in 5 patients and persisted after treatment. These abnormalities were complex and patient-specific and may be missed by group-based comparisons. The findings highlight the need for personalized white matter assessments to identify risk factors for irreversible injury and to tailor interventions aimed at improving long-term neurocognitive outcomes.

Over half of all childhood brain tumors originate in the posterior fossa.^1^ These include pilocytic astrocytoma (WHO Grade I), a slow-growing, low-grade tumor; ependymoma (WHO Grade II/III); and medulloblastoma (WHO Grade IV), the most common malignant brain tumor in childhood.^2,3^ Treatment for posterior fossa tumors typically involves surgery, followed by chemotherapy and/or radiotherapy depending on the histology of the tumor and the age of the patient. Survivors face a range of neuropsychological impairments, which impact behavior, cognition, language, and motor skills.^4–7^ Neurological outcomes are associated with white matter changes within the brain,^8–13^ but it is unclear whether injury to white matter occurs as a result of treatment, such as radiotherapy, or is already present at diagnosis, for example, secondary to hydrocephalus.^14–16^

It is possible that white matter damage observed prior to treatment can contribute to neurocognitive deficits experienced after treatment. To improve treatment strategies and patient outcomes, it is important to understand the cause of these white matter abnormalities and any potential cumulative injury over time. A noninvasive method that can be used to investigate white matter microstructure is diffusion-weighted magnetic resonance imaging (diffusion MRI), which quantifies the motion of water molecules within the brain (ie, water diffusion).

The diffusion of water molecules can be modeled using different methods. One of the most common techniques is diffusion tensor imaging (DTI), which models the magnitude and directionality of diffusion using the diffusion tensor, a symmetric 3 × 3 matrix of the magnitude of water diffusion in 3 dimensions.^17^ Water diffusion is restricted by the underlying tissue microstructure. Therefore, properties of the diffusion tensor can be used to infer information about abnormalities in the structure of white matter, such as myelination, axon organization, edema, gliosis, density, and dispersion.^18–21^ DTI parameters that can be measured include: fractional anisotropy (FA), the measurement of directionality or the degree of anisotropy of the water diffusion; mean diffusivity (MD), the magnitude of water diffusion; axial diffusivity (AD), the magnitude of diffusion parallel to the principle diffusion direction; radial diffusivity (RD), the magnitude of diffusion perpendicular to the principle diffusion direction. The most common parameter used to assess white matter microstructure in DTI studies is FA. Diffusion in white matter is expected to be more anisotropic, aligning along the white matter tracts.

Using DTI, previous studies have investigated the cause of white matter changes in posterior fossa tumor patients by comparing patient groups with different treatment types or radiation doses, analyzing the impact of tumor size or location, and exploring longitudinal changes after surgery and adjuvant treatments.^8,13–16,22–30^ However, DTI has not been used to quantify tumor-associated white matter damage in posterior fossa tumor patients prior to treatment. Variability between patients has also not been previously explored, for example, by assessing white matter in a “one-against-many” approach, which is a technique that compares an individual patient to age- and sex-matched controls (as opposed to comparing a patient group to a matched control group).

While DTI is valuable for modeling water diffusion in the brain, the model is limited by its lack of specificity. For example, changes in the anisotropy (FA) of water diffusion could result from changes in the coherence of white matter tracts and the degree to which axon bundles are aligned, or properties of the tracts themselves (axon density, diameter, or myelination). More advanced diffusion models have been developed to address limitations in the DTI model, including Neurite Orientation Dispersion Diffusion Imaging (NODDI), which enables the measurement of axon density and dispersion.^31^ NODDI may provide greater specificity than FA, where ODI has previously shown stronger correlations with histological measures of orientation dispersion than FA, which could improve sensitivity to microstructural changes^32,33^ [for a more detailed overview of NODDI clinical applications, see Kamiya et al.^34^]

In this proof-of-concept study, we sought to use DTI and NODDI to assess white matter damage in pediatric patients with posterior fossa tumors before treatment. We focus on comparing individual brain tumor patients to age- and sex-matched controls in a one-against-many approach to determine whether white matter abnormalities are present due to the tumor and its related effects.

## Methods

### Participants

This study included 8 pediatric patients with posterior fossa tumors treated at Great Ormond Street Hospital for Children (GOSH; Table 1). In all patients, surgery was their first intervention; all patients underwent MR imaging prior to surgery (Table 2). Six patients received further imaging at a time point directly after surgery (“postsurgery” or 24–72 hours after tumor resection) and/or at an early follow-up (“follow-up” or 3 months after surgical resection). Two patients had further follow-up imaging at 6–18 months after surgical resection. Approval for this study was granted as a retrospective case note review by our institutional ethical review board under 23NC04. Posterior fossa tumor patients were selected based on clinically-acquired diffusion MRI data, taken as standard clinical care.

**Table 1.** Patient and Healthy Control Participant Information

			Patient				Healthy control	
ID	Age (yrs)	Sex	Tumor type	Postsurgical scan (days)	3-month follow-up scan (days)	Further follow-up scan (days)	Age range (yrs)	Mean age (1*σ*; yrs)
PF002	8.7	F	PA	3	109		7.4–9.5	8.5 (0.5)
						547	8.4–12.3	10.1 (1.4)
PF016	7.5	F	PA	1	134		6.1–8.6	8.0 (0.8)
PF018	12.3	M	Recurrent HGG	–	79		9.8–14.6	11.8 (1.6)
PF023	14.6	F	HB	2	77		13.7–15.7	14.7 (0.6)
PF033	13.8	F	DMG	1	–		12.8–14.9	13.8 (0.6)
PF041	9.1	F	MB (SHH; div)	–	–		8.33–10.1	8.9 (0.6)
PF050	8.0	M	MB (Gr3; LCA)	–	103		6.8–9.8	8.6 (0.8)
						187	7.4–9.9	8.7 (0.7)
PF901	9.1	F	PA	–	–		8.3–10.1	9.0 (0.6)

Patient's age is given at the time of the presurgical scan. Postsurgical, 3-month follow-up, and further follow-up scan times are shown as the number of days after surgery. Tumor-type acronyms are: medulloblastoma (MB), high-grade glioma (HGG), diffuse midline glioma (DMG), pilocytic astrocytoma (PA), hemangioblastoma (HB). Medulloblastoma patients were molecular subgroups group 3 (Gr3) or SHH and had histologies of divergent (div) or large cell/anaplastic (LCA). All patients had a group of 20 age- and sex-matched healthy controls. Controls were used for multiple patients when patients were similar in age. For the 6–18 month further follow-up scans (patients PF002 and PF050), the 20 age- and sex-matched controls were updated to reflect the increase in age compared to scans at earlier time points.

**Table 2.** Treatment for the Posterior Fossa Tumor Patients

Patient	Treatment
PF002	Surgical-resection only
PF016	Surgical-resection only
PF018^*^	Surgical-resection, focal radiotherapy, chemotherapy
PF023	Surgical-resection only
PF033	Surgical-resection, focal radiotherapy
PF041	Surgical-resection, CSI + PF boost, chemotherapy
PF050^*^	Surgical-resection, CSI + PF boost, chemotherapy
PF901	Surgical-resection only

All patients had scans prior to any treatments, that is, presurgery. Patients with “*” show the patients with a scan after photon radiotherapy. Medulloblastoma patients are treated with craniospinal irradiation (CSI) with a boost to the posterior fossa (PF). Patient PF018 had previous treatment for HGG, which included focal radiotherapy administered 6 years prior to recurrence, followed by chemotherapy, with the official end of treatment approximately 4 years prior to recurrence. The treatment regime outlined above for PF018 was for the recurrence of HGG.

Healthy children were included in our analyses, for comparison to posterior fossa tumor patients, originally recruited to existing ethically approved departmental studies (REC reference numbers: 10/H0713/16, 12/LO/0442, 14/LO/0115, 15/LO/0347, 16/LO/0256; UCL Institute of Education REC approval number 1080). All control participants had no significant medical history and no radiological abnormalities were identified on MRI scans. In total, there were 102 control subjects (the female-to-male ratio was 63:39; the median age was 10.1 years with the age range of 6.1–15.7 years).

### MRI Acquisition

MRI scans were performed at GOSH on a 3.0 T MAGNETOM Prisma (Siemens Healthcare) scanner using a 20-channel head receive coil. For control subjects, either a 64- or 20-channel head receive coil was used. The clinical MRI protocols for patients included multi-shell diffusion, an axial T2-weighted turbo spin-echo, fluid-attenuated inversion recovery (FLAIR) sequences, and pre- and post-gadolinium T1-weighted acquisitions. Volumetric T1-weighted images were acquired using an MPRAGE sequence with isometric 1.0 mm voxels, relaxation time (TR) = 2300 ms, and echo time (TE) = 2.74 ms. The acquisition time was 5 minutes 21 seconds.

The multi-shell diffusion MRI sequence at our institution employed a diffusion-weighted spin-echo single shot two-dimensional echo planar imaging acquisition, with multi-banded radio frequency pulses to accelerate volume coverage along the slice direction.^35,36^ There was a multiband factor of 2 employed over 66 slices of 2 mm thickness with 0.2 mm slice gap. The diffusion gradients were applied over 2 shells at b = 1000 and 2200 s/mm^2^. There were 60 non-co-linear diffusion directions per shell with an additional 13 interleaved b_0_ (b = 0 s/mm^2^; non-diffusion weighted) images. Imaging parameters were TR = 3050 ms, TE = 60 ms, field-of-view (FOV) = 220 × 220 mm^2^, matrix size of 110 × 110, in-plane voxel resolution of 2.0 × 2.0 mm^2^, GRAPPA factor 2 and phase-encoding partial Fourier 6/8. Additionally, an identical b0 acquisition was performed to the diffusion-weighted scan but with the phase-encoding direction flipped by 180° in the anterior-posterior direction to correct artifacts related to susceptibility. The acquisition time was 7 minutes and 50 seconds for the complete multi-shell diffusion sequence.

### Image Processing and Modelling

Raw diffusion MRI files were processed using MRtrix3^37^ and Functional MRI of the Brain (FMRIB) Software Library (FSL v5.0).^38^ First, images were denoised using dwidenoise^39^ employing a brain mask to improve processing speeds. A correction was made for Gibbs ringing artifacts using mrdegibbs.^40^ Diffusion MRI and the reverse phase-encode b0 images were processed using dwifslpreproc, which uses the topup^41,42^ and eddy tools^43^ from FSL to correct for susceptibility-induced distortions, artifacts from eddy currents and movement from the participant. Lastly, B1 field inhomogeneities were corrected using dwibiascorrect.^42,44^ The diffusion tensor was calculated per voxel using FSL’s dtifit. DTI parameter maps were computed for FA, MD, AD, and RD.

The NODDI diffusion MRI modeling method was used to assess axon dispersion. This diffusion model assumes the tissue in a voxel is made of three compartments: an intracellular compartment (modeled by a set of sticks, representing the area bounded by the neurite membrane), an extracellular compartment (modeled by Gaussian anisotropic diffusion, representing the area around the neurites) and a compartment for free water or cerebrospinal fluid (CSF; modeled by isotropic Gaussian diffusion). This model estimates the density and coherence of neurites as well as the free water fraction or extent of CSF contamination. We were interested in the dispersion or coherence of the fiber tracts, known as the orientation dispersion index (ODI). Since NODDI is able to disentangle the effects of axon density from the dispersion of the axons, the ODI parameter was used to provide a more comprehensive understanding of white matter abnormalities also found using DTI.

Tissue segmentation was used to calculate the total brain volume and lateral ventricle volume of participants. Tissue segmentation was performed on T1-weighted MRI data using 5ttgen^45^ script in MRTrix3, employing the FSL algorithm. This code segments a subject’s T1-weighted MR image into five tissue types, including gray matter, subcortical gray matter, white matter, CSF, and pathological tissue.

### Tract-Based Spatial Statistics

Tract-based spatial statistics (TBSS)^46^ was employed to conduct a voxel-wise analysis of DTI parameters (FA, MD, AD, and RD) and NODDI ODI. TBSS was used to (1) compare the posterior fossa tumor patient group (*n* = 8) at the presurgical time point to a group of age- and sex-matched healthy controls (*n* = 24) to assess statistical differences in DTI parameters and (2) use a “one-against-many” approach to compare each individual patient to 20 age- and sex-matched healthy controls to assess statistical differences in DTI and ODI parameters at the different time points (presurgery, postsurgery, 3-month follow-up and any further follow-up).

For the group analysis, 3 age- and sex-matched controls were selected for each of the 8 patients to ensure a balanced representation, resulting in a control group of 24 subjects (Supplementary Methods). The control group had an age range of 7.3–14.8 years (mean age of 10.4 years; Figure S1) and the female-to-male ratio was 18:6 (75% female). The patient group at the presurgical time point had an age range of 7.5–14.6 years (mean age of 10.4 years) and the female-to-male ratio was 6:2 (75% female).

In the one-against-many approach, each patient was compared to 20 age- and sex-matched controls. Details for the control group ages for each patient can be found in Table 1. Control ages were matched so that the patient age was within 1σ of the mean control age (Figure S2).

In the TBSS analysis, the patient and controls were aligned into the Montreal Neurological Institute (MNI152) common space using FMRIB’s Nonlinear Image Registration Tool (FNIRT). A mean FA image was generated from the individual patient and control group images (thresholded at FA of 0.2), which was used to generate an FA ‘skeleton’ of white matter tracts that is study-specific, which represents the center of the white matter tracts throughout the brain. Each patient and control image was aligned to the FA skeleton, which allowed for the voxel-wise comparison of an individual patient to their control group. A voxel-wise statistical analysis was performed using a non-parametric, permutation-based approach in FSL’s randomise^47^ function. A two-sample t-test design was used to compare patients to their matched controls. For the group-level analysis, 5000 permutations were performed, while for the one-against-many analyses, 21 permutations (the maximum possible) were used. Threshold-free cluster enhancement (TFCE) was applied to improve the sensitivity of statistical detection by enhancing cluster-like structures in the data without requiring an arbitrary cluster-forming threshold. To control for multiple comparisons, results were family-wise error corrected (FWE) at *P* < .05.

To assess the quality of TBSS results and ensure significant differences in DTI parameters in the white matter skeleton between the patient and control group corresponded to voxels in the white matter tracts, the results were transformed back into the patient’s native space. A mask was generated to include the supratentorial region of the brain and exclude spurious signals that corresponded to ventricle space (Supplementary Methods).

The JHU ICBM-DTI-81 white matter labels atlas,^48^ consisting of 48 distinct white matter tracts, was used to identify clusters along the white matter skeleton with significantly different DTI parameters from the TBSS analysis.

### Lateral Ventricle Volumes

To assess the degree of patient obstructive hydrocephalus due to the presence of a posterior fossa tumor, lateral ventricle volumes were calculated from the tissue-segmented T1-weighted images (described in Image Processing and Modelling). This method used a binary mask to extract the lateral ventricles from CSF images. The binary mask template was originally produced for the Automatic Lateral Ventricle delIneatioN (ALVIN) method,^49^ based on an average of 275 CSF images from healthy subjects aged 18 to 94. The mask included the entire lateral ventricular system as well as the temporal horns. First, the mask was qualitatively assessed by applying the mask on averaged CSF maps of the patients and the controls to ensure it covered the lateral ventricles. The mask was then applied to individual patient and control CSF images and manually corrected for boundary changes when required. Lateral ventricle volumes were then calculated in mm^3^ by summing the intensity over the spatially normalized CSF segmented image. Third ventricle volumes were not calculated as it is not surrounded by white matter; the fourth ventricle was obscured by the tumor in almost all cases and was also not evaluated.

## Results

### Group Analysis

**Figure 1.**
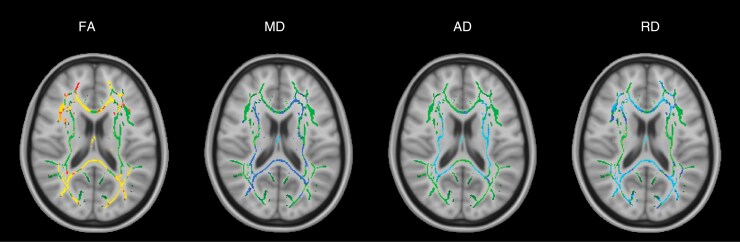
TBSS results for the group of all 8 posterior fossa tumor patients at the presurgical time point. Green denotes the white matter skeleton where voxels are not significantly different between the patient and control groups. The yellow to red color scale denotes voxels that are significantly decreased in the patient group and the light blue to blue color scale denotes voxels that are significantly increased in the patient group. Significant *P*-values for FA are <.05 (yellow values are <.01). Significant *P*-values for MD are < .05 (light blue values are <.015). AD *P*-values are <.03 (light blue values are <.005). RD *P*-values are <.05 (light blue values are <.005).

The group of 8 posterior fossa tumor patients was compared to a group of healthy controls at the presurgical time point. DTI parameters FA, MD, AD, and RD were significantly different (*P*-value < .05) between the patient and control group in many white matter tracts throughout the brain presurgery (Figure 1). There was widespread decreased FA and increased MD, AD, and RD in regions corresponding to the corpus callosum (body, splenium, and genu), fornix, corona radiata, and posterior thalamic radiations. The FA decrease corresponded to 36.1% of the white matter skeleton. Diffusivity increases corresponded to 18.6% (MD), 16.4% (AD), and 35.9% (RD) of the white matter skeleton.

### Individual Analysis

#### Presurgical time point.—

**DTI** Out of the 8 patients individually compared to 20 age- and sex-matched controls, 5 patients exhibited significant changes in FA prior to surgery. Three of these patients had “widespread” changes to white matter, which we define as changes in FA across >5% of the total white matter skeleton and >15% of the labeled white matter tracts identified in the JHU ICBM-DTI-81 atlas. The other 2 patients had changes to <5% of the white matter skeleton, defined as “localized” changed to white matter.

**Figure 2.**
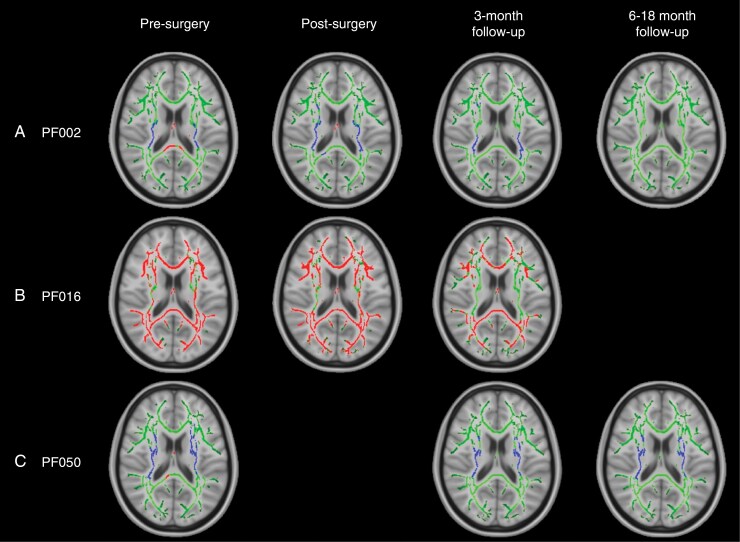
TBSS results for individual patients with widespread, significant changes in FA, including pilocytic astrocytoma patients PF002 (A) and PF016 (B) and medulloblastoma patient PF050 (C). Green corresponds to the white matter skeleton where voxels are not significantly different between the individual patient and group of controls. Red voxels correspond to areas of the white matter skeleton that are significantly decreased in the patient (*P*-value < .05), and blue voxels are significantly increased in the patient (*P*-value < .05). From left to right, the image timeline is from presurgery, postsurgery, follow-up at 3 months and a further follow-up at 6–12 months (as in Table 1).

The pattern of widespread FA changes can be split into two categories:

1. An increase in FA corresponding to the periventricular white matter (PVWM), that is, internal capsule and corona radiata (patients PF002 and PF050, Figure 2, A and C).
2. A decrease in FA (patient PF016, Figure 2B) in all white matter tracts.

These patients also had significant changes in diffusivity throughout the white matter skeleton (details of MD, AD, and RD are provided in Supplementary Results). Diffusivity changes were more complex than FA:

1. PF002: MD, AD, and RD (Figure S5) showed a pattern of increased values near the ventricles, surrounded by white matter with decreased values. In PVWM, specifically the internal capsule and corona radiata, both MD and RD were decreased and there were areas of both increased and decreased AD. The posterior thalamic radiations exhibited decreased FA, MD, and AD with increased RD. The fornix was the only area to have decreased FA with increased MD, AD, and RD.
2. PF016: There were widespread increases in MD and RD (Figure S8). AD showed a more complex pattern similar to PF002 with increased values in PVWM, surrounded by decreased values distal to the ventricles.
3. PF050: AD was primarily increased, particularly in PVWM, corresponding to the internal capsule and corona radiata, while RD was decreased (Figure S11). MD was mainly unchanged. The fornix deviated from the other areas of white matter with decreased FA and increased MD, AD, and RD.

Furthermore, there were 2 patients with localized decreases in FA at the presurgical time point, and there were no changes in diffusivity parameters for these patients. PF018 had decreased FA in the body and genu of the corpus callosum and the corona radiata (1.2% of the total white matter skeleton) and PF901 had decreased FA in the left corona radiata (0.3% of the total white matter skeleton). However, patient PF018 had recurrent HGG and had previously received focal radiotherapy, so the presurgical treatment for this patient could have been due to earlier treatment.

**Figure 3.**
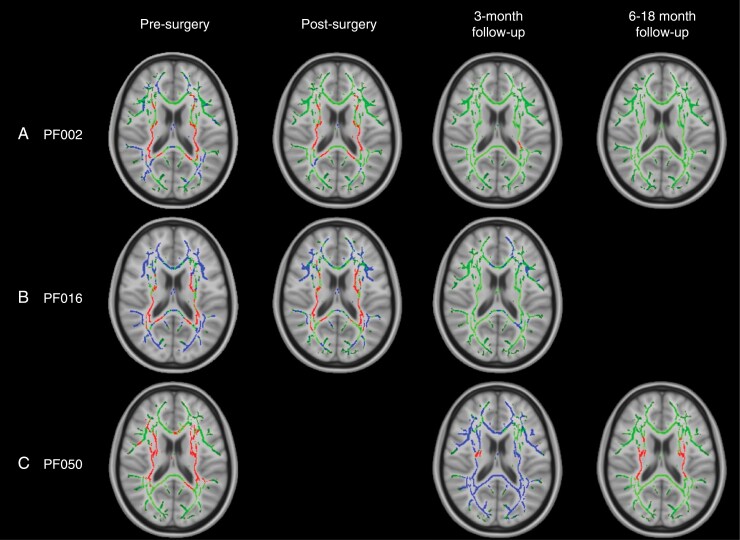
TBSS results for individual patients with widespread, significant changes in ODI, including pilocytic astrocytoma patients PF002 (A) and PF016 (B) and medulloblastoma patient PF050 (C). Green corresponds to the white matter skeleton where voxels are not significantly different between the individual patient and group of controls. Red voxels correspond to areas of the white matter skeleton that are significantly decreased in the patient (*P*-value < .05), and blue voxels are significantly increased in the patient (*P*-value < .05). From left to right, the image timeline is from presurgery, postsurgery, follow-up at 3 months and a further follow-up at 6–12 months (as in Table 1).

**NODDI ODI** Prior to surgery, only patients PF002, PF016, and PF050 with widespread FA changes across the white matter skeleton were found to have significant alterations in ODI compared to their control groups (Figure 3). These changes were found in 2 patterns:

1. A widespread decrease in ODI corresponding to PVWM, that is, internal capsule and corona radiata (patients PF002 and PF016), surrounded by an increase in ODI.
2. A widespread decrease in ODI in PVWM only (patient PF050).

#### Longitudinal changes.—

**DTI** Over time, DTI parameters did not fully return to normal in patients with presurgical changes (Figure 2). At the postsurgical time point, patients PF002, PF016, and PF050 generally had DTI parameters that were relatively similar or potentially improved compared to their presurgical scan. At follow-up, there was evidence of improvement but results were mixed:

1. PF002: FA returned to normal values over an 18-month period after surgery, specifically in PVWM (internal capsule and corona radiata), which originally had elevated FA. However, there was a decrease in diffusivity (MD, AD, and RD) that became more widespread through the white matter skeleton at 3 months and 18 months after surgery. More details are provided in Supplementary Results (Figures S5-S7).
2. PF016: FA remained significantly decreased across many white matter tracts from the presurgical time point to the 3-month follow-up. Diffusivity parameters mainly returned to normal values. AD was only increased in the fornix and RD was increased in the fornix, body, and splenium of the corpus callosum and areas corresponding to PVWM (internal capsule and corona radiata). More details are provided in Supplementary Results (Figures S8-S10).
3. PF050: At 3 months postsurgery (after radiotherapy), FA remained significantly increased in PVWM, corresponding to the internal capsule, corona radiata, and external capsule. The only change in diffusivity corresponded to increased RD in the fornix, which also showed decreased FA. At 6 months postsurgery (3 months after CSI), MD was decreased in the left hemisphere. RD was decreased in PVWM, similar to the presurgical scan. More details are provided in Supplementary Results (Figures S11-S13).

In addition to the 3 patients with changes at the presurgical time point, there were 2 other patients, PF018 and PF033, with decreased FA postsurgery. In patient PF018, there was a change in the number of voxels with decreased FA between the presurgical time point and 3-month follow-up, increasing from 1.2% to 10.8% of the white matter skeleton. This corresponded to the corpus callosum (genu, body, and splenium), corona radiata, and posterior thalamic radiations. Patient PF033 was found to have decreased FA in 15.9% of the voxels in the postsurgical scan, which corresponded to the corpus callosum (genu, body, and splenium), internal capsule, fornix, corona radiata, posterior thalamic radiations, superior longitudinal fasciculus, inferior fronto-occipital fasciculus, and uncinate fasciculus.

**NODDI ODI** After surgery, only patients with presurgical changes in ODI were found to have longitudinal changes in this parameter, including PF002, PF016, and PF050. Over time, patients PF002 and PF016 had ODI parameters that began to return to normal after 3–18 months. A different trend was observed in patient PF050. Initially, PF050 had decreased ODI in PVWM. In the 3-month follow-up (after radiotherapy), there was a widespread increase in ODI in all white matter tracts. At the 6-month follow-up scan (3 months post-radiotherapy), there was a decrease in ODI in PVWM that resembled the presurgical TBSS analysis.

#### Lateral ventricle volumes.—

**Figure 4.**
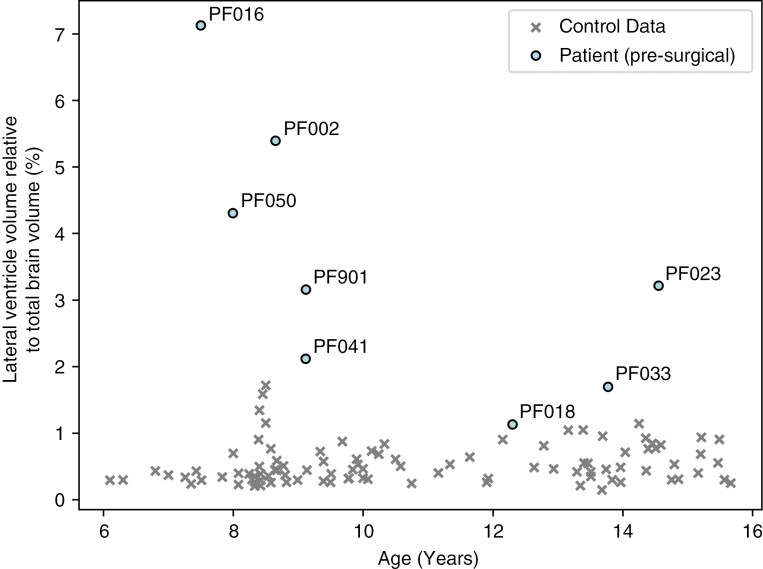
Comparison of the lateral ventricle volumes relative to the total brain volume (%) and age (years) of the patients at the presurgical time point and all controls.

All patients had enlarged lateral ventricles (*P*-value < .05) when compared to their age- and sex-matched controls (Figure 4). The 3 patients with the most enlarged lateral ventricles (lateral ventricle volumes >4% of the total brain volume) correspond to the 3 patients with the most widespread changes in FA.

Over time, the lateral ventricles decreased in size for most of the patients following surgery (PF002, PF016, PF023, PF050), but remained significantly enlarged for patients PF002, PF016, and PF050 compared to their control groups. In patients PF018 and PF033, the ventricles grew larger after surgery (PF018: 1.1% to 1.8%; PF033: 1.7% to 1.9% of the total brain volume).

## Discussion

Our study is the first to demonstrate, using diffusion imaging, significant changes to supratentorial white matter caused by the presence of an infratentorial tumor prior to treatment. Using a one-against-many comparison, we found that observed white matter abnormalities are complex and patient-dependent, indicating that white matter changes may be missed using analysis techniques that group posterior fossa brain tumor patients together for comparison with a control group.

In the posterior fossa patient group analysis of presurgical images, we found widespread decreased FA and increased diffusivity (MD, AD, and RD) throughout numerous white matter tracts compared to the healthy control group. At this time point, all patients were found to have significantly enlarged lateral ventricles relative to total brain volume, likely due to hydrocephalus caused by the tumor obstructing the fourth ventricle. These DTI abnormalities could indicate that there was increased diffusion in patient white matter and the diffusion was less directional (more isotropic). This was consistent with edema or damage to white matter, for example, reduced coherence of white matter tracts, decreased fiber density, or damage to white matter integrity.^50,51^ Obstructive hydrocephalus can cause a rupture of the ventricular ependymal lining due to increasing intraventricular pressure. CSF can then move into the extracellular spaces leading to increased interstitial fluid, that is, edema.^52,53^ Decreased FA and/or increased diffusivity were consistent with past studies that have used DTI to assess the white matter in groups of posterior fossa tumor patients, both postsurgery but before adjuvant treatment^16^ and post-treatment.^8,13–15,22–30^

Even though white matter abnormalities were present in the presurgical group analysis, the one-against-many technique was able to highlight the heterogeneity of white matter abnormalities in individual posterior fossa tumor patients, which were not captured when comparing the patient group to the control group. Prior to surgery, we found that 5 out of 8 patients—who had different tumor types, including high-grade glioma, medulloblastoma, and pilocytic astrocytoma—had evidence of supratentorial white matter injury using the one-against-many approach. Three of the patients, including PF002, PF016, and PF050, showed widespread changes in FA in >5% of the white matter skeleton. These patients had the most enlarged ventricle volumes at the presurgical time point, corresponding to lateral ventricles >4 % of the total brain volume (>10σ above the healthy control mean). Other patients with DTI changes prior to surgery included PF018, a high-grade glioma patient, and PF901, a pilocytic astrocytoma patient. Both patients had decreased FA in small, localized areas with no changes in diffusivity. While these patients had enlarged ventricles, the degree of ventricular enlargement was not as extreme as the patients with widespread DTI changes. This finding suggests that hydrocephalus potentially impacts the brain white matter microstructure prior to surgery.

Compression effects due to hydrocephalus were also seen in the analysis of the axonal dispersion using NODDI ODI. In patients, PF002, PF016, and PF050, the pattern of decreased ODI in PVWM prior to surgery may be an indicator of tissue compression caused by enlarged ventricles, where we expect compression to cause a decrease in axon dispersion (or an increase in tract coherence) in PVWM. In patients PF002 and PF016, the area surrounding PVWM was seen to have decreased coherence (increased ODI), which is potentially related to the greater size of the ventricles.

The widespread white matter changes found before surgery were observed to persist over time, particularly FA. The widespread presurgical changes in FA were observed in different patterns, including a decrease in FA throughout supratentorial white matter (PF016: pilocytic astrocytoma) and an increase in FA corresponding to periventricular white matter (PF002: pilocytic astrocytoma; PF050: medulloblastoma). These FA patterns are consistent with studies that have used DTI to understand white matter microstructure in patients with hydrocephalus (eg, Assaf et al.,^54^ Ben-Sira et al.,^55^ Air et al.,^56^ Hattingen et al.,^57^ Osuka et al.,^58^ Yuan et al.^59^). Increased FA in the periventricular region may be the result of the compression of these white matter tracts due to enlarged lateral ventricles,^54,60,61^ where fibers that are homogeneously aligned will be packed together more closely. Decreased FA may be indicative of microstructural white matter damage or diffuse edema caused by increased pressure in the ventricles that disrupts the ventricular ependymal lining. Chronic tissue compression from hydrocephalus has previously been found to result in tissue degeneration, including demyelination, axonal loss, gliotic change, loss of neuronal transmission, and higher packing of white matter fibers,^62–64^ and there is evidence that the largest ventricles lead to worse PVWM destruction and deficits in motor and cognitive function.^65^ This could lead to cumulative damage to white matter, for example, when adjuvant treatment involves radiotherapy. To improve the outcomes of patients, pretreatment imaging could be considered during individual treatment planning and risk evaluation to avoid further injury to white matter or to minimize the volume of white matter damage.

In addition to studying the effects of hydrocephalus, NODDI may be beneficial for interpreting short and long-term white matter abnormalities due to radiotherapy. For example, the medulloblastoma patient PF050 was treated with surgical resection, CSI with a posterior fossa boost, and chemotherapy. While the TBSS analysis of the ODI parameter originally showed evidence of increased white matter coherence (decreased ODI) in PVWM prior to surgery, at the 3-month follow-up scan there was evidence of widespread decreased coherence (increased ODI) across all white matter tracts. This time point corresponded to the end of radiotherapy. Decreased coherence in white matter can indicate axonal disorganization or increased extra-axonal space.^66^ This is consistent with early radiotherapy effects, such as inflammation, which is expected to contribute to demyelination over time caused by the apoptosis of oligodendrocytes.^67^ At the 6-month follow-up scan (3 months after treatment), the TBSS analysis of ODI returned to a similar pattern as the presurgical analysis (ie, decreased dispersion at PVWM). There was also new evidence of white matter abnormalities in the DTI analysis at this time point, indicated by decreased MD in the left hemisphere white matter tracts (Figure S11). These patterns differed from those observed in a high-grade glioma patient (PF018) who received focal radiotherapy, suggesting that radiation dose and field size may influence the extent and nature of white matter changes detected by DTI and NODDI. In PF018, there was evidence of worsening white matter injury after radiotherapy, with decreased FA affecting 1.2% of the white matter skeleton presurgery and expanding to 10.8% post-treatment. However, unlike in PF050, there were no detectable changes in diffusivity or NODDI parameters.

The one-against-many technique is a novel approach in diffusion MRI, enabling a more individualized understanding of how white matter integrity is impacted over time in pediatric brain tumor patients. A benefit of the one-against-many approach is that it allows for the variability between white matter abnormalities in patients of similar ages and with the same tumor type to be explored. For example, we compare the female pilocytic astrocytoma patients PF002, PF016, and PF901 who were 8.7, 7.5, and 9.1 years in age at the time of surgery, respectively. These patients had no further treatment after surgical resection of the tumor. The patients showed different white matter abnormalities, likely due to the effects of hydrocephalus, evidenced by an increase in FA in PF002 in PVWM, a decrease in FA in PF016 and both patients had evidence of compression effects in ODI (increased coherence in PVWM surrounded by decreased coherence further from the ventricles). PF901 was found to have a localized decrease in FA in the left corona radiata and no change in ODI or diffusivity. The differences between these patients are likely due to the ventricle sizes, where PF016 had the most enlarged ventricles compared to total brain volume (7.1%) compared to PF002 (5.4%) and PF901 (3.2%). Over time, both PF002 and PF016 have FA and ODI values that began to return to normal over a 3–18-month period, but there were differences in how diffusivity parameters changed over time. MD values across the white matter skeleton showed large-scale significant decreases compared to control values that affected more of the white matter skeleton in the later timescales 3–18 months after surgery, which could be due to gliosis (Figure S8). This may indicate that the effects of hydrocephalus on PF002 are different and more long-lasting than PF016 or PF901.

The ability to conduct the one-against-many analysis quickly—approximately 13 minutes for FA and an additional 6 minutes for other diffusivity parameters—makes it feasible to incorporate into clinical workflows. This has important implications confirming that brain injury can occur pretreatment and distant to the tumor location, as evidenced by the abnormal supratentorial white matter seen in childhood posterior fossa tumors. This may allow tailoring of future therapeutic or rehabilitation strategies, as white matter abnormalities detected early could inform adjustments in treatment plans to mitigate long-term neurocognitive deficits. Prior studies have used DTI (lower FA) to show that white matter abnormalities observed postsurgery but prior to adjuvant treatment correlate with later cognitive decline,^16^ and tumor size is associated with abnormalities (increased diffusivity) in specific white matter pathways,^17^ further highlighting the potential long-term impact of early white matter changes.^68^

Early prediction of white matter vulnerability in pediatric brain tumor patients could enable more personalized treatment strategies. This may include modifications to radiotherapy treatment plans,^69^ selection of patients for pharmacological interventions^70,71^ and early planning of rehabilitation strategies such as group physical exercise.^72–75^ Cognitive follow-up will be essential in future studies to correlate pretreatment and longitudinal white matter changes with cognitive outcomes. Future work could (1) explore the differences in microstructure and motor and cognitive function in patients with infratentorial tumors to better understand the impact of obstructive hydrocephalus on long-term outcomes, (2) understand risk factors for white matter injury that persists despite effective treatment of ventriculomegaly and (3) identify the contribution of adjuvant therapy for individual patients.

Our study has several limitations. The sample size of this study is small, though it is comparable to other exploratory studies involving the use of DTI to understand microstructural changes in white matter in posterior fossa tumor patients at postsurgical and/or post-treatment time periods.^8,10,27^ With larger patient numbers, the underlying cause of DTI changes prior to treatment could be further investigated, including the impact of different tumor types on white matter abnormalities. Furthermore, the cumulative effects of the tumor and treatment modalities, which may lead to worse neurocognitive outcomes, could be explored. Additionally, one of the challenges of a one-against-many approach is the need to acquire at least 20 age- and sex-matched healthy controls for each patient on the same MRI machine. In this study, the control groups for each patient are not evenly distributed in age; however, care was taken to ensure the age of the patient was within 1σ of the mean control age. Discrepancies in the spread of ages in the control group for each patient could affect the sensitivity of the TBSS analysis depending on how closely the available control data matched the patient’s age.

In conclusion, diffusion MRI can be used to detect white matter abnormalities before treatment in posterior fossa brain tumor patients. This knowledge may contribute to disentangling the causes of white matter damage in the brain, either from the tumor, associated hydrocephalus, or from treatments such as radiotherapy. Furthermore, the one-against-many approach shows that pretreatment white matter changes are patient-dependent, which may have a bearing on the ultimate treatment response and neurocognitive outcomes of patients. This approach underlines the variation and individuality of white matter injury in children with a posterior fossa tumor and has the potential to define risk factors for irreversibility despite post-treatment reduction in ventricular size. This may have implications for understanding white matter vulnerability in individual children, tailoring adjuvant therapy to reduce risk and early planning of rehabilitation strategies. These white matter abnormalities may, therefore, need to be considered in treatment planning for individual patients to improve outcomes and prevent cumulative white matter damage.

## Supplementary material

Supplementary material is available online at *Neuro-Oncology Advances* (https://academic.oup.com/noa).

vdaf053_suppl_Supplementary_Figures_S1-S13

## Data Availability

Data will be made available upon reasonable request to the authors.

## References

[1] Pollack IF. Brain tumors in children. N Engl J Med. 1994;331(22):1500–1507. 7969301 10.1056/NEJM199412013312207

[2] Louis DN , Perry A, Wesseling P, et al The 2021 WHO classification of tumors of the central nervous system: A summary. Neuro-Oncology. 2021;23(8):1231–1251. 34185076 10.1093/neuonc/noab106 PMC8328013

[3] Louis DN , Perry A, Reifenberger G, et al The 2016 World Health Organization classification of tumors of the central nervous system: A summary. Acta Neuropathol. 2016;131(6):803–820. 27157931 10.1007/s00401-016-1545-1

[4] Robinson KE , Fraley CE, Pearson MM, Kuttesch JF, Compas BE. Neurocognitive late effects of pediatric brain tumors of the posterior fossa: A quantitative review. J Int Neuropsychol Soc. 2013;19(1):44–53. 23095276 10.1017/S1355617712000987

[5] Fiorillo A , Rinaldi M, Foggia L. Gait analysis in children treated by surgery followed by adjuvant therapy for posterior fossa tumors. Acta Neurol Belg. 2010;110(4):306–310. 21305859

[6] Wolfe KR , Madan-Swain A, Kana RK. Executive dysfunction in pediatric posterior fossa tumor survivors: A systematic literature review of neurocognitive deficits and interventions. Dev Neuropsychol. 2012;37(2):153–175. 22339228 10.1080/87565641.2011.632462 PMC3730812

[7] Mabbott DJ , Penkman L, Witol A, Strother D, Bouffet E. Core neurocognitive functions in children treated for posterior fossa tumors. Neuropsychology. 2008;22(2):159–168. 18331158 10.1037/0894-4105.22.2.159

[8] Khong PL , Kwong DLW, Chan GCF, Sham JST, Chan FL, and Ooi GC. Diffusion-tensor imaging for the detection and quantification of treatment- induced white matter injury in children with medulloblastoma: A pilot study. Technical report, 2003. https://www.ncbi.nlm.nih.gov/pmc/articles/PMC8148675/. PMC8148675 12695214

[9] Khong PL , Leung LHT, Fung ASM, et al White matter anisotropy in post-treatment childhood cancer survivors: Preliminary evidence of association with neurocognitive function. J Clin Oncol 2006;24(6):884–890. 16484697 10.1200/JCO.2005.02.4505

[10] Mabbott DJ , Noseworthy MD, Bouffet E, Rockel C, Laughlin S. Diffusion tensor imaging of white matter after cranial radiation in children for medulloblastoma: Correlation with IQ. Neuro-Oncology. 2006;8(3):244–252. 16723629 10.1215/15228517-2006-002 PMC1871945

[11] Aukema EJ , Caan MWA, Oudhuis N, et al White matter fractional anisotropy correlates with speed of processing and motor speed in young childhood cancer survivors. Int J Radiat Oncol Biol Phys. 2009;74(3):837–843. 19117694 10.1016/j.ijrobp.2008.08.060

[12] Rueckriegel SM , Bruhn H, Thomale UW, Driever PH. Cerebral white matter fractional anisotropy and tract volume as measured by MR imaging are associated with impaired cognitive and motor function in pediatric posterior fossa tumor survivors. Pediatr Blood Cancer 2015;62:1252–1258. 25850573 10.1002/pbc.25485

[13] Moxon-Emre I , Bouffet E, Taylor MD, et al Vulnerability of white matter to insult during childhood: Evidence from patients treated for medulloblastoma. J Neurosurg Pediatr. 2016;18(1):29–40. 27015518 10.3171/2016.1.PEDS15580

[14] Law N , Bouffet E, Laughlin S, et al Cerebello–thalamo–cerebral connections in pediatric brain tumor patients: Impact on working memory. Neuroimage. 2011;56(4):2238–2248. 21473922 10.1016/j.neuroimage.2011.03.065

[15] McEvoy SD , Lee A, Poliakov A, et al Longitudinal cerebellar diffusion tensor imaging changes in posterior fossa syndrome. NeuroImage Clin 2016;12:582–590. 27689022 10.1016/j.nicl.2016.09.007 PMC5031477

[16] Glass JO , Ogg RJ, Hyun JW, et al Disrupted development and integrity of frontal white matter in patients treated for pediatric medulloblastoma. Neuro Oncol. 2017;19(10):1408–1418. 28541578 10.1093/neuonc/nox062 PMC5596166

[17] Basser PJ , Mattiello J, Le Bihan D. MR diffusion tensor spectroscopy and imaging. Biophys J. 1994;66(1):259–267. 8130344 10.1016/S0006-3495(94)80775-1 PMC1275686

[18] Wang S , Wu EX, Qiu D, et al Longitudinal diffusion tensor magnetic resonance imaging study of radiation-induced white matter damage in a rat model. Cancer Res. 2009;69(3):1190–1198. 19155304 10.1158/0008-5472.CAN-08-2661

[19] Song SK , Sun SW, Ramsbottom MJ, et al Dysmyelination revealed through MRI as increased radial (but unchanged axial) diffusion of water. Neuroimage. 2002;17(3):1429–1436. 12414282 10.1006/nimg.2002.1267

[20] Beaulieu C. The basis of anisotropic water diffusion in the nervous system – a technical review. NMR Biomed. 2002;15(7-8):435–455. 12489094 10.1002/nbm.782

[21] Basser PJ , Pierpaoli C. Microstructural and physiological features of tissues elucidated by quantitative-diffusion-tensor MRI. J Magn Reson B. 1996;111(3):209–219. 8661285 10.1006/jmrb.1996.0086

[22] Perreault S , Ramaswamy V, Achrol AS, et al Yeom. MRI surrogates for molecular subgroups of medulloblastoma. Am J Neuroradiol 2014;35(7):1263–1269. 24831600 10.3174/ajnr.A3990 PMC4819007

[23] Palmer SL , Glass JO, Li Y, et al White matter integrity is associated with cognitive processing in patients treated for a posterior fossa brain tumor. Neuro Oncol. 2012;14(9):1185–1193. 22898373 10.1093/neuonc/nos154 PMC3424215

[24] Scantlebury N , Bouffet E, Laughlin S, et al White matter and information processing speed following treatment with cranial-spinal radiation for pediatric brain tumor. Neuropsychology. 2016;30(4):425–438. 26752125 10.1037/neu0000258

[25] Hua C , Merchant TE, Gajjar A, et al Brain tumor therapy-induced changes in normal-appearing brainstem measured with longitudinal diffusion tensor imaging. Int J Radiat Oncol Biol Phys. 2012;82(5):2047–2054. 21664060 10.1016/j.ijrobp.2011.03.057 PMC3181276

[26] Uh J , Merchant TE, Li Y, et al Differences in brainstem fiber tract response to radiation: A longitudinal diffusion tensor imaging study. Int J Radiat Oncol Biol Phys. 2013;86(2):292–297. 23474114 10.1016/j.ijrobp.2013.01.028 PMC3646932

[27] Qiu D , Leung LHT, Kwong DLW, Chan GCF, Khong PL. Mapping radiation dose distribution on the fractional anisotropy map: Applications in the assessment of treatment-induced white matter injury. Neuroimage. 2006;3(1):109–115. 10.1016/j.neuroimage.2005.12.007 16448821

[28] Khong PK , Leung LHT, Chan GCF, et al White matter anisotropy in child-hood medulloblastoma survivors: Association with neurotoxicity risk factors. Radiology. 2005;236(2):647–652. 16040920 10.1148/radiol.2362041066

[29] Qiu D , Kwong DLW, Chan GCF, Leung LHT, Khong PL. Diffusion tensor magnetic resonance imaging finding of discrepant fractional anisotropy between the frontal and parietal lobes after whole-brain irradiation in child-hood medulloblastoma survivors: Reflection of regional white matter radiosensitivity. Int J Radiat Oncol Biol Phys. 2007;69(3):846–851. 17544593 10.1016/j.ijrobp.2007.04.041

[30] Palmer SL , Reddick WE, Glass JO, et al Regional white matter anisotropy and reading ability in patients treated for pediatric embryonal tumors. Brain Imaging Behav 2010;4(2):132–140. 20502994 10.1007/s11682-010-9092-1 PMC3521043

[31] Zhang H , Schneider T, Wheeler-Kingshott CA, Alexander DC. NODDI: Practical in vivo neurite orientation dispersion and density imaging of the human brain. Neuroimage. 2012;61(4):1000–1016. 22484410 10.1016/j.neuroimage.2012.03.072

[32] Grussu F , Schneider T, Tur C, et al Neurite dispersion: a new marker of multiple sclerosis spinal cord pathology? Ann Clin Transl Neurol. 2017;4(9):663–679. 28904988 10.1002/acn3.445 PMC5590517

[33] Schilling KG , Janve V, Gao Y, et al Histological validation of diffusion MRI fiber orientation distributions and dispersion. Neuroimage. 2018;165:200–221. 29074279 10.1016/j.neuroimage.2017.10.046 PMC5732036

[34] Kamiya K , Hori M, Aoki S. NODDI in clinical research. J Neurosci Methods. 2020;346:108908. 32814118 10.1016/j.jneumeth.2020.108908

[35] Setsompop K , Cohen-Adad J, Gagoski BA, et al Improving diffusion MRI using simultaneous multi-slice echo planar imaging. Neuroimage. 2012;63(1):569–580. 22732564 10.1016/j.neuroimage.2012.06.033 PMC3429710

[36] Setsompop K , Gagoski BA, Polimeni JR, et al Blipped-controlled aliasing in parallel imaging for simultaneous multislice echo planar imaging with reduced g-factor penalty. Magn Reson Med. 2012;67(5):1210–1224. 21858868 10.1002/mrm.23097 PMC3323676

[37] Tournier JD , Smith R, Raffelt D, et al Mrtrix3: A fast, flexible and open software framework for medical image processing and visualisation. Neuroimage. 2019;202:116137. 31473352 10.1016/j.neuroimage.2019.116137

[38] Jenkinson M , Beckmann CF, Behrens TEJ, Woolrich MW, Smith SM. FSL. Neuroimage. 2012;62(2):782–790. 21979382 10.1016/j.neuroimage.2011.09.015

[39] Veraart J , Novikov DS, Christiaens D, et al Denoising of diffusion MRI using random matrix theory. Neuroimage. 2016;142:394–406. 27523449 10.1016/j.neuroimage.2016.08.016 PMC5159209

[40] Kellner E , Dhital B, Kiselev VG, Reisert M. Gibbs-ringing artifact removal based on local subvoxel-shifts. Magn Reson Med. 2016;76(5):1574–1581. 26745823 10.1002/mrm.26054

[41] Andersson JLR , Skare S, Ashburner J. How to correct susceptibility distortions in spin-echo echo-planar images: Application to diffusion tensor imaging. Neuroimage. 2003;20(2):870–888. 14568458 10.1016/S1053-8119(03)00336-7

[42] Smith SM , Jenkinson M, Woolrich MW, et al Advances in functional and structural MR image analysis and implementation as FSL. Neuroimage. 2004;23:S208–S219. 15501092 10.1016/j.neuroimage.2004.07.051

[43] Andersson JLR , Sotiropoulos SN. An integrated approach to correction for off-resonance effects and subject movement in diffusion MR imaging. Neuroimage. 2016;125:1063–1078. 26481672 10.1016/j.neuroimage.2015.10.019 PMC4692656

[44] Zhang Y , Brady M, Smith S. Segmentation of brain MR images through a hidden Markov random field model and the expectation-maximization algorithm. IEEE Trans Med Imaging. 2001;20(1):45–57. 11293691 10.1109/42.906424

[45] Smith RE , Tournier JD, Calamante F, Connelly A. Anatomically-constrained tractography: Improved diffusion MRI streamlines tractography through effective use of anatomical information. Neuroimage. 2012;62(3):1924–1938. 22705374 10.1016/j.neuroimage.2012.06.005

[46] Smith SM , Jenkinson M, Johansen-Berg H, et al Tract-based spatial statistics: Voxelwise analysis of multi-subject diffusion data. Neuroimage. 2006;31(4):1487–1505. 16624579 10.1016/j.neuroimage.2006.02.024

[47] Winkler AM , Ridgway GR, Webster MA, Smith SM, Nichols TE. Permutation inference for the general linear model. Neuroimage. 2014;92:381–397. 24530839 10.1016/j.neuroimage.2014.01.060 PMC4010955

[48] Wakana S , Caprihan A, Panzenboeck MM, et al Reproducibility of quantitative tractography methods applied to cerebral white matter. Neuroimage. 2007;36(3):630–644. 17481925 10.1016/j.neuroimage.2007.02.049 PMC2350213

[49] Kempton MJ , Underwood TSA, Brunton S, et al A comprehensive testing protocol for MRI neuroanatomical segmentation techniques: Evaluation of a novel lateral ventricle segmentation method. Neuroimage. 2011;58(4):1051–1059. 21835253 10.1016/j.neuroimage.2011.06.080 PMC3551263

[50] Mori S , Zhang J. Principles of diffusion tensor imaging and its applications to basic neuroscience research. Neuron. 2006;51(5):527–539. 16950152 10.1016/j.neuron.2006.08.012

[51] Sundgren PC , Dong Q, Gmez-Hassan D, et al Diffusion tensor imaging of the brain: Review of clinical applications. Neuroradiology. 2004;46(5):339–350. 15103435 10.1007/s00234-003-1114-x

[52] Ulug AM , Truong TN, Filippi CG, et al Diffusion imaging in obstructive hydrocephalus. Am J Neuroradiol. 2003;24(6):1171–1176. 12812949 PMC8148995

[53] Nehring SM , Tadi P, Tenny S. Cerebral Edema. 2023. https://pubmed.ncbi.nlm.nih.gov/30725957/30725957

[54] Assaf Y , Ben-Sira L, Constantini S, Chang LC, Beni-Adani L. Diffusion tensor imaging in hydrocephalus: Initial experience. Am J Neuroradiol. 2006;27(8):1717–1724. 16971621 PMC8139798

[55] Ben-Sira L , Goder N, Bassan H, et al Clinical benefits of diffusion tensor imaging in hydrocephalus. J Neurosurg Pediatr. 2015;16(2):195–202. 25978534 10.3171/2014.10.PEDS13668

[56] Air EL , Yuan W, Holland SK, et al Longitudinal comparison of pre- and postoperative diffusion tensor imaging parameters in young children with hydrocephalus. J Neurosurg Pediatr. 2010;5(4):385–391. 20367345 10.3171/2009.11.PEDS09343

[57] Hattingen E , Jurcoane A, Melber J, et al Diffusion tensor imaging in patients with adult chronic idiopathic hydrocephalus. Neurosurgery. 2010;66(5):917–924. 20404696 10.1227/01.NEU.0000367801.35654.EC

[58] Osuka S , Matsushita A, Yamamoto T, et al Evaluation of ventriculomegaly using diffusion tensor imaging: Correlations with chronic hydrocephalus and atrophy. J Neurosurg. 2010;112(4):832–839. 19698041 10.3171/2009.7.JNS09550

[59] Yuan W , Mangano FT, Air EL, et al Anisotropic diffusion properties in infants with hydrocephalus: A diffusion tensor imaging study. Am J Neuroradiol. 2009;30(9):1792–1798. 19661167 10.3174/ajnr.A1663 PMC7051526

[60] Eriksson SH , Rugg-Gunn FJ, Symms MR, Barker GJ, Duncan JS. Diffusion tensor imaging in patients with epilepsy and malformations of cortical development. Brain. 2001;124(3):617–626. 11222460 10.1093/brain/124.3.617

[61] Wieshmann UC. Diffusion tensor imaging demonstrates deviation of fibres in normal appearing white matter adjacent to a brain tumour. J Neurol Neurosurg Psychiatry 2000;68(4):501–503. 10727488 10.1136/jnnp.68.4.501 PMC1736891

[62] Bergstrom K , Thuomas KA, Ponten U, et al Magnetic resonance imaging of brain tissue displacement and brain tissue water contents during progressive brain compression. An experimental study in dogs. Acta Radiol Suppl. 1986;369:350–352. 2980493

[63] Obrien JP , Mackinnon SE, MacLean AR, et al A model of chronic nerve compression in the rat. Ann Plast Surg. 1987;19(5):430–435. 3688790 10.1097/00000637-198711000-00008

[64] Siegal T , Siegal TZ, Sandbank U, et al Experimental neoplastic spinal cord compression: Evoked potentials, edema, prostaglandins, and light and electron microscopy. Spine. 1987;12(5):440–448. 3629394 10.1097/00007632-198706000-00004

[65] Del Bigio MR , Wilson MJ, Enno T. Chronic hydrocephalus in rats and humans: White matter loss and behavior changes. Ann Neurol. 2003;53(3):337–346. 12601701 10.1002/ana.10453

[66] Kamiya K , Hori M, Aoki S. NODDI in clinical research. J Neurosci Methods. 2020;346:108908. 32814118 10.1016/j.jneumeth.2020.108908

[67] Makale MT , McDonald CR, Hattangadi-Gluth JA, Kesari S. Mechanisms of radiotherapy-associated cognitive disability in patients with brain tumours. Nat Rev Neurol. 2016;13(1):52–64. 27982041 10.1038/nrneurol.2016.185 PMC5805381

[68] Drabek-Maunder ER , Mankad K, Aquilina K, et al Using diffusion MRI to understand white matter damage and the link between brain microstructure and cognitive deficits in paediatric medulloblastoma patients. Eur J Radiol. 2024;177:111562. 38901074 10.1016/j.ejrad.2024.111562

[69] Sienna J , Kahalley LS, Mabbott D, et al Proton therapy mediates dose reductions to brain structures associated with cognition in Children With Medulloblastoma. Int J Radiat Oncol Biol Phys. 2024;119(1):200–207. 38040059 10.1016/j.ijrobp.2023.11.035 PMC11023754

[70] Ayoub, R., Ruddy, R.M., Cox, E., Oyefiade, A., Derkach, D., et al Assessment of cognitive and neural recovery in survivors of pediatric brain tumors in a pilot clinical trial using metformin. Nat Med. 2020;26(8):1285–1294. doi: https://doi.org/10.1038/s41591-020-0985-2. 32719487 PMC8176964

[71] Verity SJ , Halliday G, Hill RM, Ryles J, Bailey S. Methylphenidate improves cognitive function and health-related quality of life in survivors of childhood brain tumours. Neuropsychol Rehabil. 2024;34(1):133–153. 36580420 10.1080/09602011.2022.2157446

[72] Wurz A , McLaughlin E, Lategan C, Ellis K, Culos-Reed SN. Synthesizing the literature on physical activity among children and adolescents affected by cancer: evidence for the international Pediatric Oncology Exercise Guidelines (iPOEG). Transl Behav Med. 2021;11:699–708. 33538309 10.1093/tbm/ibaa136 PMC8033595

[73] Riggs L , Piscione J, Laughlin S, et al Exercise training for neural recovery in a restricted sample of pediatric brain tumor survivors: a controlled clinical trial with crossover of training versus no training. Neuro Oncol. 2017;19(3):440–450. 27555603 10.1093/neuonc/now177 PMC5464296

[74] Szulc-Lerch KU , Timmons BW, Bouffet E, et al Repairing the brain with physical exercise: Cortical thickness and brain volume increases in long-term pediatric brain tumor survivors in response to a structured exercise intervention. NeuroImage: Clin. 2018;18:972–985. 29876282 10.1016/j.nicl.2018.02.021 PMC5987848

[75] Cox E , Bells S, Timmons BW, et al A controlled clinical crossover trial of exercise training to improve cognition and neural communication in pediatric brain tumor survivors. Clin Neurophysiol. 2020;131(7):1533–1547. 32403066 10.1016/j.clinph.2020.03.027

